# Resistin Promotes the Expression of Vascular Endothelial Growth Factor in Ovary Carcinoma Cells

**DOI:** 10.3390/ijms14059751

**Published:** 2013-05-07

**Authors:** Li Pang, Yi Zhang, Yu Yu, Shulan Zhang

**Affiliations:** 1 Department of Obstetrics and Gynecology, Shengjing Hospital Affiliated to China Medical University, Shenyang 110004, Liaoning, China; E-Mails: doctorpang16@163.com (L.P.); abc112113114@163.com (Y.Y.); 2 Department of Gynecology, the First Affiliated Hospital of China Medical University, Shenyang 110001, Liaoning, China; E-Mail: syzi960@yahoo.com

**Keywords:** resistin, vascular endothelial growth factor (VEGF), Sp1, phosphoinositide 3-kinase (PI3K)/Akt

## Abstract

Resistin is a novel hormone that is secreted by human adipocytes and mononuclear cells and is associated with obesity, insulin resistance and inflammation. Recently, resistin has been postulated to play a role in angiogenesis. Here, we investigated the hypothesis that resistin regulates ovary carcinoma production of vascular endothelial growth factor (VEGF) and the angiogenic processes. We found that in human ovarian epithelial carcinoma cells (HO-8910), resistin (10–150 ng/mL) enhanced both VEGF protein and mRNA expression in a time- and concentration-dependent manner, as well as promoter activity. Furthermore, resistin enhanced DNA-binding activity of Sp1 with VEGF promoter in a PI3K/Akt-dependent manner. PI3K/Akt activated by resistin led to increasing interaction with Sp1, triggering a progressive phosphorylation of Sp1 on Thr453 and Thr739, resulting in the upregulation of VEGF expression. In an *in vitro* angiogenesis system for endothelial cells (EA.hy926) co-cultured with HO-8910 cells, we observed that the addition of resistin stimulated endothelial cell tube formation, which could be abolished by VEGF neutralizing antibody. Our findings suggest that the PI3K/Akt-Sp1 pathway is involved in resistin-induced VEGF expression in HO-8910 cells and indicates that antiangiogenesis therapy may be beneficial treatment against ovarian epithelial carcinoma, especially in obese patients.

## 1. Introduction

Ovarian cancer is one of the most common causes of death from all cancers among women and the leading cause of death from gynecological malignancies. Ovarian epithelial carcinoma is a common malignant ovarian neoplasm with poor five-year survival rate (less than 30%). Many factors regulate the rapid growth of ovarian epithelial carcinoma. The autocrine secretion hypothesis proposes that, as a result of oncogene activation, neoplastic cells can produce certain growth factors to promote the tumor proliferation or angiogenesis [1].

Dysregulated angiogenesis is involved in pathological conditions such as ischemic heart diseases, cancer, diabetes, or chronic inflammation [2]. Tumor vessels develop by sprouting or intussusception from pre-existing vessels. Circulating endothelial precursors, shed from the vessel wall or mobilized from the bone marrow, can also contribute to tumor angiogenesis [3,4]. Various molecules involved in these different mechanisms of vascular growth [5]. Among these, vascular endothelial growth factor (VEGF) has a predominant role. The angiogenic activity of VEGF is tightly regulated by gene dosage [6]. VEGF promotes growth, proliferation, migration, formation of new blood vessels [7] and survival of endothelial cells. VEGF regulation can occur at both transcriptional and post-transcriptional levels in a cell-specific manner.

Various signals trigger tumor angiogenesis, including metabolic stress such as obesity, mechanical stress, immune/inflammatory response, and genetic mutations [8]. Adipokines could be important for the development of obesity-related diseases such as diabetes, cardiovascular diseases, and cancer [9]. Although the literature to date focusing on obesity and prognosis of ovarian cancer has been inconclusive, a recent meta-analysis reported a positive relationship between early adulthood BMI and mortality among patients with ovarian cancer [10]. Resistin, a peptide hormone mainly secreted by adipose tissue, has been suggested to be involved in the pathogenesis of insulin resistance and so may link obesity to diabetes [11]. In humans, macrophages, similar in many aspects to adipocytes, also have been reported as the predominant source of circulating resistin [12]. Resistin, also known as FIZZ3 (found in the inflammatory zone), is closely related to inflammation [13] and cancer [14]. Although resistin promotes *in vitro* angiogenesis of human endothelial cells directly [15,16], however, the role of resistin in tumor angiogenesis still remains unclear.

In the present study, we investigated whether VEGF expression could be induced by resistin in human ovarian epithelial carcinoma cells and explored the mediating mechanism and possible significance.

## 2. Results and Discussion

### 2.1. VEGF Expression and Production in Ovarian Epithelial Carcinoma Cells Was Induced by Resistin

**Figure 1:**
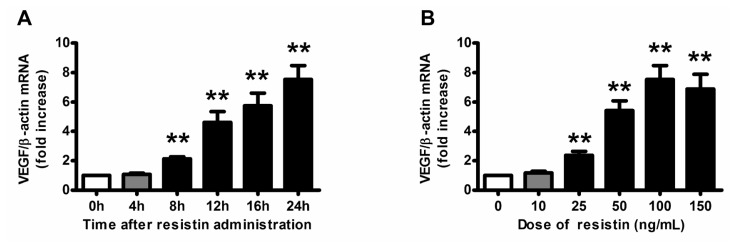

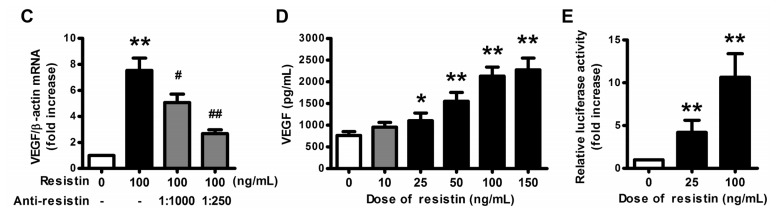
VEGF expression and production in ovarian epithelial carcinoma cells was induced by resistin. (**A**) Time course of 100 ng/mL resistin treatment on VEGF mRNA levels in HO-8910 cells; (**B**) Dose-response effect of 24-h resistin treatment on VEGF mRNA levels in HO-8910 cells; (**C**) Attenuation of resistin-induced VEGF expression by anti-resistin neutralizing antibody in HO-8910 cells. Cells were pretreated with or without anti-resistin neutralizing antibody (1:1000 or 1:250) for 1 h, then incubated in the presence of resistin 100 ng/mL for 24 h. Relative mRNA levels were normalized to that of untreated cells; (**D**) Dose-response effect of 24-h resistin treatment on VEGF peptide levels in the medium of HO-8910 cells; (**E**) Effects of resistin on VEGF promoter (pLuc 1) activity in HO-8910 cells. Cells were transfected with pLuc 1 together with control plasmid with use of jetPEI reagent. After transfection for 24 h, cells were stimulated with 100 ng/mL resistin or PBS, then luciferase activity was measured. Relative luciferase activity was normalized with respect to the activity in untreated cells. The results are representative of four independent experiments performed in triplicate. Data are means ± SEM. *p < 0.05; **p < 0.01 *vs.* untreated cells; ^#^*p* < 0.05, ^##^*p* < 0.01 *vs.* resistin treatment alone.

Exposure of HO-8910 cells, the human ovarian epithelial carcinoma cell line, to resistin (100 ng/mL) induced VEGF mRNA expression in a time-dependent manner. Elevation in VEGF mRNA level occurred as early as 8 h and remained increased for up to 24 h (Figure 1A). The resistin treatment (10–150 ng/mL) for 24 h also caused a concentration-dependent increase in VEGF mRNA expression (Figure 1B), with maximal induction of 7.6-fold found with 100 ng/mL of resistin. Therefore, in our subsequent experiments, the resistin dosage we choose was 100 ng/mL, which is consistent with other reports in human choriocarcinoma cells [16] and in vascular smooth muscle cells [17]. Neutralizing antibody of resistin (1:1000 and 1:250) significantly attenuated the resistin-induced VEGF mRNA expression (Figure 1C), which indicates that resistin-stimulated VEGF expression depends on resistin immune activity. Additionally, resistin also induced the VEGF peptide synthesis and secretion into the culture media (Figure 1D). Furthermore, we transfected the VEGF promoter fragment (pLuc 1) into HO-8910 cells and found that resistin induced luciferase activity significantly (Figure 1E). Taken together, resistin upregulates VEGF expression and production in ovarian epithelial carcinoma cells through transcription pathway.

### 2.2. PI3K/Akt Pathway Is Activated upon Resistin Stimulation

**Figure 2:**
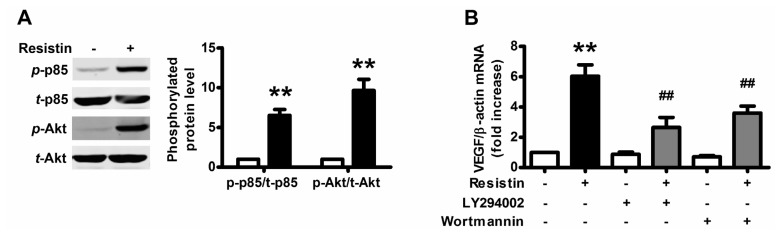

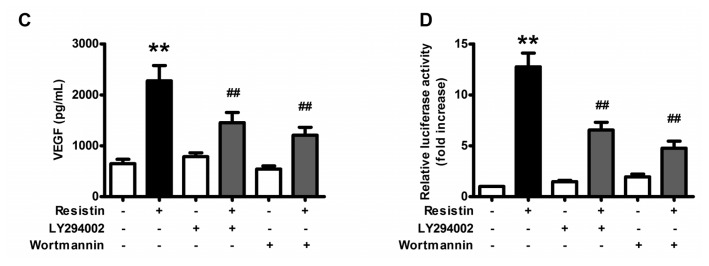
PI3K/Akt pathway was activated upon resistin stimulation. (**A**) Effects of resistin on PI3K/Akt pathway activation in HO-8910 cells. Cells were incubated with 100 ng/mL resistin for 1 h and whole-cell lysates underwent Western blotting to detect the phosphorylation of p85 subunit of PI3K and Akt (Ser 473); (**B**–**D**) Attenuation of resistin-induced VEGF mRNA expression (**B**), peptide secretion (**C**) and promoter activity (**D**) by LY294002 and wortmannin in HO-8910 cells. Cells were pretreated with (+) or without (−) LY294002 (20 μM) and wortmannin (2 μM) for 1 h, then incubated in the presence (+) or in the absence (−) of resistin 100 ng/mL for 24 h. Relative VEGF mRNA levels were normalized with respect to the levels for untreated cells. The basal promoter activity of each test plasmid is indicated as luciferase activity normalized by each internal control activity (PRL-TK). Relative luciferase activity was normalized with respect to the activity in untreated cells. The results are representative of four independent experiments performed in triplicate. Data are means ± SEM. **p < 0.01 *vs.* untreated cells, ^##^*p* < 0.01 *vs.* resistin treatment alone.

Although the resistin receptor has not been identified, phosphoinositide 3-kinase (PI3K)/Akt signal, downstream of multiple cell-surface receptor types and implicated in angiogenesis, was presumed to be activated. To investigate whether the activation of PI3K/Akt is involved in the resistin response, we used Western blotting to detect the phosphorylation of p85 subunit of PI3K and Akt (Ser 473), the activated form of PI3K/Akt pathway. After resistin stimulation for 1 h, the phosphorylation of p85 subunit of PI3K and Akt (Ser 473) was significantly elevated (Figure 2A).

To investigate if the PI3K/Akt pathway was involved in resistin-induced VEGF expression, LY294002 (20 μM) and wortmannin (2 μM), the specific inhibitors of the PI3K/Akt pathway, were used. As shown in Figure 2B, LY294002 and wortmannin significantly blocked resistin-induced increment in the expression of VEGF mRNA. Similar results were also observed in peptides secretion (Figure 2C) and promoter activity (Figure 2D). Therefore, resistin upregulates VEGF expression through the PI3K/Akt pathway.

### 2.3. Localization of the Resistin Regulatory Element in the VEGF Gene Promoter

**Figure 3:**
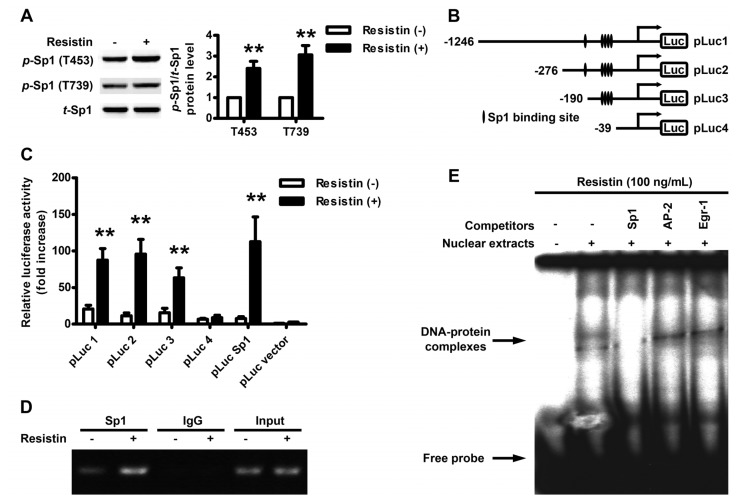
Sp1 acted on the motif of VEGF promoter. (**A**) Effects of resistin on Sp1 activation in HO-8910 cells. Cells were incubated with 100 ng/mL resistin for 2 h and whole-cell lysates underwent Western blotting to detect the phosphorylation of Sp1 at either Thr-453 or Thr-739; (**B**) Scheme of the series of plasmid constructs containing dissected fragments of VEGF promoter; (**C**) Relative luciferase activity in transient transfection assays using the series of plasmid constructs (schematically shown in **B**) and pSp1-luciferase reporter. Relative luciferase activity was normalized with respect to the activity of PGL3-basic in untreated cells. Data are mean ± SEM from four independent triplicated experiments; (**D**) Chromatin immunoprecipitation of VEGF promoter complexes. HO-8910 cells underwent immunoprecipitation with anti-Sp1 antibody or control IgG. Immunoprecipitated chromatin fragments were amplified by PCR with specific primers targeting the predicted Sp1 binding motifs within the VEGF promoter; (**E**) Effects of various competitors in EMSA of resistin-stimulated HO-8910 cells. Competitors, including Sp1, AP-2 and Egr-1 consensus sequences, were added to the reaction mixture at a 100-fold molar excess to labeled probe, the VEGF promoter fragment containing predicted Sp1 binding motifs. Lane 2 contained no competitor. Nuclear extracts were prepared from HO-8910 cells and underwent EMSA. Cells were pre-incubated with 100 ng/mL resistin for 24 h.

It was reported that the high GC-rich motifs in the proximal regions of VEGF promoter are regulated by transcriptional factor Sp1; Sp1 phosphorylation at Thr-453 and Thr-739 was implicated in extracellular signal-regulated kinase (ERK)-mediated VEGF gene transcription. Therefore, the phosphorylation status of Sp1 following resistin treatment of HO-8910 cells was then examined. Western blot analysis revealed that resistin treatment significantly increased the phosphorylation of Sp1 at either Thr-453 or Thr-739 (Figure 3A). To identify the cis-regulatory element responsible for this resistin effect, the luciferase activity of cells transfected with a series of promoter 5′-deletion constructs was examined. The results showed that resistin-induced luciferase activity was observed using a construct containing Sp1-binding region (pLuc 1–3) or the pLuc Sp1-luciferase reporter, but not using a shorter construct without containing Sp1-binding region (pLuc 4) (Figure 3C). ChIP-PCR analysis confirmed a *bona fide* interaction of Sp1 to these corresponding response elements within the VEGF promoter (Figure 3D).

Because there are also AP-2 (−79 to −72) and Egr-1 (−77 to −70) binding sites inside the multiple Sp1 binding sites (−94 to −52), we then detected the DNA-protein binding activity by EMSA using nuclear extracts of resistin-stimulated HO-8910 cells. VEGF promoter fragment containing multiple Sp1 biding sites was used as a probe. As shown in Figure 3E, the specific bands representing DNA-protein binding activity were detected after resistin stimulation and these bindings could be abolished by the competitive Sp1 consensus oligonucleotide, but not by AP-2 and Egr-1 consensus oligonucleotides. Thus, these data suggest that Sp1 binding sites are critical for the resistin-induced VEGF gene expression in HO-8910 cells.

### 2.4. Resistin Induces PI3K/Akt-dependent Phosphorylation of Sp1

**Figure 4:**
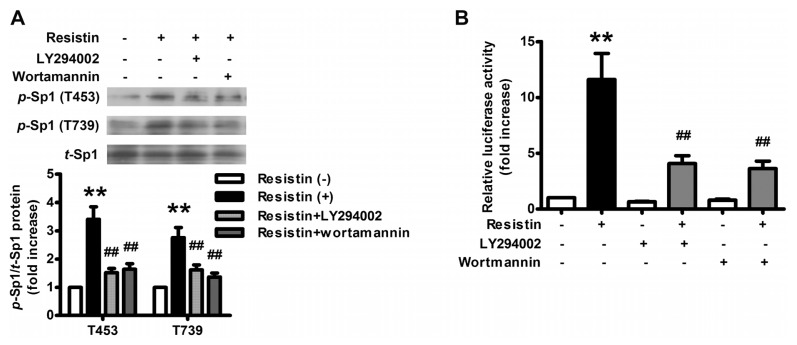
Activated PI3K/Akt upon resistin interacted with Sp1 and activated it through phosphorylation. (**A**) Blockage of resistin-induced Sp1 activation by LY294002 and wortmannin in HO-8910 cells. Cells were pretreated with (+) or without (−) LY294002 (20 μM) and wortmannin (2 μM) for 1 h, then incubated in the presence (+) or in the absence (−) of resistin 100 ng/mL for 2 h. The top panel is a representative blot obtained from three independent experiments. The lower panel represents the summary results of Sp1 phosphorylation at either Thr-453 or Thr-739. Relative phosphorylated Sp1 protein levels were normalized to that of untreated cells; (**B**) Attenuation of resistin-induced Sp1 binding activity by LY294002 and wortmannin in HO-8910 cells. Cells were transfected with pSp1-luciferase reporter together with control plasmid. After being pretreated with (+) or without (−) LY294002 (20 μM) and wortmannin (2 μM) for 1 h, cells were incubated in the presence (+) or in the absence (−) of resistin 100 ng/mL for 24 h. Data are mean ± SEM from four independent triplicated experiments. The basal binding activity of each test plasmid is indicated as luciferase activity normalized by each internal control activity (PRL-TK). Relative luciferase activity was normalized with respect to the activity in untreated cells. **p < 0.01 *vs.* untreated cells, ^##^*p* < 0.01 *vs.* resistin treatment alone.

To confirm the role of PI3K/Akt pathway in the transcriptional regulation of VEGF by resistin, the phosphorylation status of Sp1 following resistin treatment was then examined. Western blot analysis showed that resistin-induced phosphorylation of Sp1 at either Thr-453 or Thr-739 was abolished by pre-treatment with LY294002 and wortmannin (Figure 4A), as was the activity of the pLuc Sp1-luciferase reporter (Figure 4B). These results indicated that resistin-induced Sp1 activation is PI3K/Akt-dependent.

### 2.5. VEGF Mediates Resistin-Induced Angiogenesis of Ovary Carcinoma *in Vitro*

**Figure 5:**
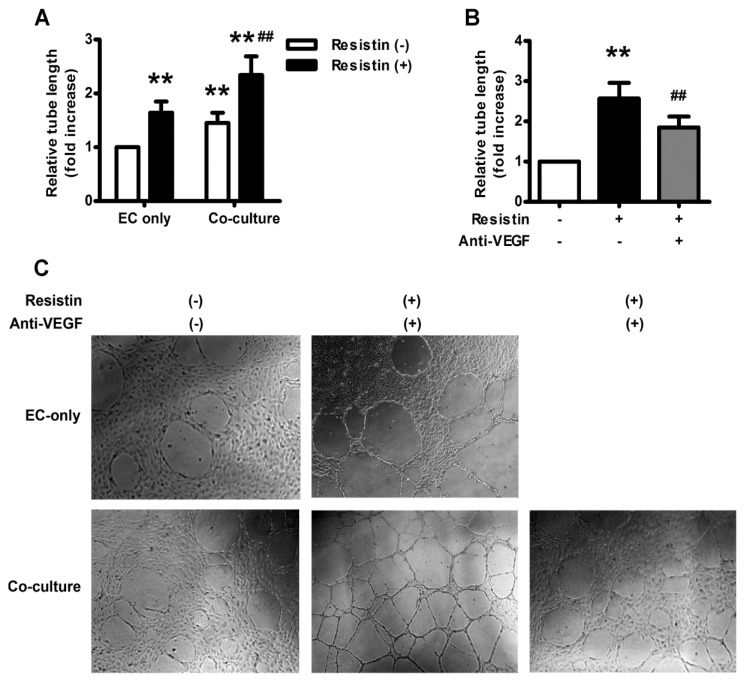
HO-8910 cells-derived VEGF mediated resistin-induced angiogenesis of EA.hy926 cells. (**A**) Capillary-like tube formation assay of EA.hy926 cells co-cultured with HO-8910 cells under resistin (100 ng/mL) stimulation for 18 h. The total length of capillary-like tubes was measured and normalized with resistin-untreated EA.hy926 cells. Data are mean ± SEM from three independent experiments. ** *p* < 0.01 *vs.* resistin-untreated EA.hy926 cells only, ^##^*P* < 0.01 *vs.* resistin-treated EA.hy926 cells; (**B**) Blockage of VEGF by neutralizing antibody retarded capillary-like tube formation ability of EA.hy926 cells co-cultured with resistin-stimulated HO-8910 cells. The total length of capillary-like tubes was measured and normalized with controls. Data are mean ± SEM from three independent experiments. **p < 0.01 *vs.* untreated cells, ^##^*p* < 0.01 *vs.* resistin treatment alone; (**C**) Representative microscopic view of capillary-like tube formation in EA.hy926 cells co-cultured with HO-8910 cells under resistin (100 ng/mL) stimulation without or with VEGF neutralizing antibody treatment.

To investigate whether resistin could affect the angiogenic property of endothelial cells, we performed a capillary-like tube formation assay on Matrigel, the most commonly used method for *in vitro* angiogenesis. EA.hy926 cells were co-cultured with HO-8910 cells pretreated with or without resistin (100 ng/mL) for 18 h. Quantitative analyses revealed that resistin treatment alone could increase the total length of capillary-like tubes in EA.hy926 cells significantly, which is further enhanced in EA.hy926 cells co-cultured with HO-8910 cells (Figure 5A,C), but abolished by VEGF neutralizing antibody (Figure 5B,C), indicating that resistin might promote tumor angiogenesis through autocrine or paracrine effects of tumor-derived factors, such as VEGF.

### 2.6. Discussion

The present study demonstrated that resistin can induce the expression of VEGF through the PI3K/Akt-Sp1 pathway in human ovarian epithelial carcinoma cell line HO-8910 cells and subsequently contribute to cancer development by promoting tumor angiogenesis. This conclusion is supported by the following observations: (1) Resistin upregulates VEGF mRNA expression in HO-8910 cells in a time-and concentration-dependent manner; (2) Resistin simulates VEGF secretion in HO-8910 cells; (3) Resistin enhances VEGF promoter activity; (4) Transcription factor Sp1 mediates the effects of resistin; (5) Inhibition of PI3K/Akt blocks the effects of resistin on Sp1, therefore attenuates the upregulation of VEGF expression; (6) Blocking of HO-8910 cell-derived VEGF inhibits resistin-stimulated capillary-like tube formation of EA.hy926 cells. To the best of our knowledge, this is the first report demonstrating the involvement of multiple signaling pathways in the resistin-mediated modulation of VEGF expression in ovarian epithelial carcinoma.

Ovarian carcinoma is the fourth most common cause of cancer death among women in the United States, although it is the 12th most common cause of cancer death among women in China—the mortality is more than 70% within five years. A relation between ovarian cancer mortality and obesity has been previously established, as obesity impacts ovarian cancer mortality by influencing tumor biology. Significant differences were found in the risk of ovarian cancer progression and ovarian cancer-related mortality associated with increasing BMI in a fairly “dose-dependent” fashion [18]. Adipose tissue is now broadly recognized as a genuine endocrine organ secreting several bioactive adipocytokines, which regulate physiological and pathological processes, such as insulin sensitivity and resistance, inflammation, immunity, and angiogenesis [19–21]. Resistin is a novel adipocytokine secreted from adipocytes and monocytes [22], being implicated in inflammatory processes including atherosclerosis, non-alcoholic fatty liver disease, and malignancies [17,22]. Interestingly, recent epidemiologic studies have indicated that serum resistin is significantly elevated in patients with breast [23], gastric [24], colorectal [25] and endometrial adenocarcinoma [26]; high resistin expression is associated with a more malignant clinicopathological status, as well as adverse prognosis [27]. Generally, resistin is considered to promote the proliferation, adhesion and invasion of tumor cells [16,22,28–30]. Our study also gave a new clue that resistin promoted tumor angiogenesis of ovarian epithelial carcinoma through the autocrine/paracrine effect of upregulated VEGF (Figure 1), since VEGF neutralizing antibody could abolish of capillary-like tube formation of EA.hy926 cells co-cultured with HO-8910 cells (Figure 5).

VEGF, one of the most important angiogenic switch molecules, exerts great influence on the behaviors of endothelial cells, including migration, proliferation, and differentiation [31,32]. VEGF plays a central role in tumor angiogenesis, drugs have been developed to target VEGF, such as bevacizumab, the monoclonal antibody directed at VEGF. In 2004, bevacizumab was approved by the Food and Drug Administration (FDA) for use in metastatic colorectal cancer. Subsequently, bevacizumab was granted approval in advanced non-small cell lung cancer, glioblastoma multiforme, and renal cell carcinoma [33]. Therefore, our study indicated that antiangiogenesis therapy such as bevacizumab may also be beneficial treatment against ovarian epithelial carcinoma, especially in obese patients.

Many factors participated in the transcriptional regulation of VEGF. The proximal GC-rich region in the VEGF promoter contains binding sites for AP-2, Egr-1, WT1, NF-κB and SP1/SP3 transcription factors, while distal enhancer sites bind AP-1 and HIF-1α [34–37]. Among these, the transcriptional factor Sp1 has been attested to regulate the expression of thousands of genes implicated in an array of cellular processes, such as cell growth, proliferation, angiogenesis and endothelial cell function in response to physiologic and pathological stimuli [38–41]. Sp1 binds directly to the GC box element of DNA to activate or repress gene transcription [42]. The high GC-rich motifs in the proximal regions of the VEGF promoter are regulated by Sp1 [43]. It was reported that in mouse mammary tumor cells that: leptin upregulated VEGF expression through Sp1 [44]; low-power laser irradiation activated Sp1 to promote VEGF expression and vascular endothelial cell proliferation [42]; and phosphorylation of Sp1 at Thr-453/Thr-739 is crucial for heme oxygenase-1/carbon monoxide-induced VEGF expression in myocytes [45]. In our study, we demonstrated that resistin upregulated VEGF expression through the phosphorylation of Sp1 at Thr-453/Thr-739 (Figure 3), which is consistent with previous reports. It is reported that Egr-1 is also crucial in regulating VEGF expression [46], and there are also AP-2 and Egr-1 binding sites inside the multiple Sp1 binding sites within VEGF promoter. However, the specific DNA-protein binding bands could only be abolished by the competitive Sp1 consensus oligonucleotide, but not by AP-2 and Egr-1 consensus oligonucleotides (Figure 3E), indicating that Sp1 binding sites are critical for the resistin-induced VEGF gene expression in ovarian epithelial carcinoma. Further investigations are still needed to determine whether other transcriptional factors, such as NF-κB and HIF-1α, are involved in resistin-induced VEGF regulation.

Sp1-dependent transcription can be regulated by a variety of signals through alternating Sp1 abundance, DNA binding activity, and/or transactivation activity which is influenced by a series of posttranslational modifications [42], especially phosphorylation via protein kinases including c-Jun NH2-terminal kinase 1 (JNK1) [47], p38 kinase [45], extracellular signal-regulated kinase (ERK) [42] and PI3K/Akt [48,49]. The PI3K/Akt pathway enhanced recruitment of human ovarian cancer endothelial progenitor cells and promotes angiogenesis [50]. Moreover, the expression of a constitutively active form of PI3K in ovarian cells results in increased expression of VEGF, thus facilitating their expansion by upregulating angiogenesis [51]. In our study, we found that resistin-induced Sp1 activation is PI3K/Akt-dependent (Figure 4) and inhibition of PI3K/Akt pathway significantly blocked resistin-induced increment in the expression of VEGF (Figure 2). Taken together, our data support the notion that resistin-induced VEGF via the PI3K/Akt-Sp1 signaling pathway may be responsible for increased angiogenesis in ovarian carcinoma.

In summary, this study showed a novel connection among resistin, VEGF and ovarian cancer. Additional studies are needed to better define how these interactions are initiated and regulated and to demonstrate whether similar effects play a role *in vivo*.

## 3. Material and Methods

### 3.1. Chemicals and Reagents

HO-8910 cell, a human ovarian epithelial carcinoma cell line, was purchased from American Type Culture Collection (ATCC, Manassas, VA, USA). RPMI1640 medium was purchased from Hyclone Co. (Logan, UT, USA). Recombinant human resistin and anti-resistin neutralizing antibody was purchased from Phoenix Pharmaceuticals (Belmont, CA, USA). LY294002 and wortmannin were purchased from CalBiochem (La Jolla, CA, USA). Anti-human VEGF neutralizing antibody was purchased from Oncogene Science (Cambridge, MA, USA). Mouse-anti-β-actin antibodies and purified rabbit IgG were purchased from Santa Cruz Biotechnology (Santa Cruz, CA, USA). All other antibodies were purchased from Cell Signaling Technology (Beverly, MA, USA). JetPEI reagent was purchased from Polyplus-transfection® SA (Illkirch, France). The Sp1 reporter assay kit was purchased from Stratagene (La Jolla, CA, USA). Sp1, AP-2 and Egr-1 consensus oligonucleotides were purchased from Promega (Madison, WI, USA). All other chemicals and drugs were purchased from Sigma Chemical (St. Louis, MO, USA).

### 3.2. Cell Culture

HO-8910 cells, a human ovarian epithelial carcinoma cell line, were cultured in RPMI1640 containing 10% FBS and penicillin/streptomycin (100 U/mL) in a humidified 37 °C incubator. When confluent, cells were treated with resistin (10–150 ng/mL) for 4–24 h. For the inhibition experiments, cells were pretreated with the LY294002 and wortmannin for 1 h prior to stimulation with resistin at 100 ng/mL for 24 h.

### 3.3. RNA Extraction and Quantitative Real Time PCR Analysis

Total RNAs were isolated using Trizol reagent according to the manufacturer’s instructions. Total RNA (2 μg) was reverse-transcribed using reverse transcription system (Promega, Madison, WI, USA). One microliter of the reaction mixture was subjected to PCR. The amount of PCR products formed in each cycle was evaluated on the basis of SYBR Green I fluorescence. The forward and reverse PCR primers were: human VEGF 5′-CTT GCC TTG CTG CTC TAC C-3′ and 5′-CAC ACA GGA TGG CTT GAA G-3′ (NM_001171623.1); human β-actin 5′-ATC TGG CAC CAC ACC TTC -3′ and 5′-AGC CAG GTC CAG ACG CA-3′ (NM_001101.3). All amplification reactions were performed using the Mx3000 Multiplex Quantitative PCR System (Stratagene, La Jolla, CA, USA) under the following conditions: 95 °C for 5 min, followed by 40 cycles at 95 °C for 30 s, 58 °C for 30s and 72 °C for 30 s. Results were analyzed with Stratagene Mx3000 software and VEGF mRNA levels were normalized with respect to the levels of β-actin in each sample. PCR reactions were performed in duplicate and each experiment was repeated for 3–5 times.

### 3.4. Quantitation of VEGF by ELISA

HO-8910 cells (4 × 10^4^/well) were cultured to 100% confluence in 24-well plate. The cells were then treated as described above. VEGF production from culture supernatants was quantitatively measured by using a human VEGF Quantikine ELISA kit (R & D Systems, Minneapolis, MN, USA) according to the manufacturer’s instructions.

### 3.5. Preparation of Cytosolic Proteins and Western Blot Analysis

Following treatment, the cells were packed by centrifuging the cells for 3 min at 200× *g*, and homogenized in ice-cold fractionation buffer (50 mM Tris-HCl, pH 7.4, 1 mM EDTA, 150 mM NaCl, 1% Triton X-100, 1 mM PMSF, 10 μg/mL leupeptin, 10 μg/mL pepstatin A, 10 μg/mL aprotinin, 1 mM sodium orthovanadate [Na_3_VO_4_], 10 mM sodium pyrophosphate [Na_4_P_2_O_7_] and 50 mM sodium fluoride [NaF]). The cell lysate was incubated on ice for 15 min and then centrifuged at 20,000× *g* for 30 min at 4 °C. The cytosolic fraction was collected and subjected to SDS-PAGE with a 10% running gel. Protein concentrations were determined by BCA Protein Assay Kit (PIERCE, Rockford, IL, USA). The proteins were transferred to a polyvinylidene fluoride membrane. The membrane was incubated successively with 5% bovine serum albumin in tris-tween buffered saline (TTBS) at room temperature for 1 h, with different first antibodies at 4 °C for 12 h and then with horseradish peroxidase-labeled second antibody for 1 h. After each incubation, the membrane was washed extensively with TTBS, and the immunoreactive band was detected with ECL-detecting reagents (PIERCE, Rockford, IL, USA).

### 3.6. Plasmid Construction and Transient Transfection Assays

The human VEGF promoter-luciferase hybrid genes were constructed as follows. A series of fragments (positions −1246, −276, −190, −39 to +316) were amplified from peripheral blood monoclonal human genomic DNA and subcloned into the pGL3basic luciferase reporter vector (Promega, Madison, WI, USA) termed pLuc 1–4 (Figure 4B).

After treatment, HO-8910 cells in 24-well plates were transfected by jetPEI reagent with each plasmid DNA, followed by 24-h incubation and the harvesting. Cells were then lysed and luciferase activity was measured. The amounts of DNA used for transfection were 0.5 μg of a test fusion gene and 10 ng of an internal control, PRL-TK (Promega, Madison, WI, USA). Expression of reporter genes and PRL-TK was determined with the Dual-Luciferase™ Reporter Assay System (Promega, Madison, WI, USA).

### 3.7. Preparation of Nuclear Proteins and Electrophoretic Mobility Shift Assay (EMSA)

Nuclear proteins were extracted with the use of NE-PER reagents (Pierce, Rockford, IL, USA). VEGF promoter fragment containing Sp1 biding sites (−276 to −24) was amplified by PCR and end-labeled by DIG gel shift assay system (Roche, Penzberg, Germany) as detection probe. For competition reaction, nuclear extracts were treated with unlabeled oligonucleotide for 10 min prior to the binding reaction. The DNA-protein binding reaction was performed at room temperature for 15 min in a 20 μL reaction mixture. The following oligonucleotides were used as competitors: Sp1, AP-2 and Erg-1 consensus sequence.

### 3.8. Chromatin Immunoprecipitation (ChIP) and PCR Analysis

Following treatment, cells underwent immunoprecipitation with anti-Sp1. Immunoprecipitated chromatin fragments were amplified by PCR. The primers used for amplification of the VEGF promoter were: sense, 5′-CCT GCC CCC TTC AAT ATT CCT -3′; and antisense, 5′-ATA TCA AAT TCC AGC ACC GAG C -3′ [52].

### 3.9. Capillary-Like Tube Formation Assay

The formation of capillary-like structures by EA.hy926 cells on Matrigel (Becton Dickinson, Bedford, MA) was studied. HO-8910 cells were cultured in lower chambers of 24-well transwell culture plates and were pretreated with or without resistin (100 ng/mL) for 24 h. Upper chambers were coated with Matrigel according to the manufacturer’s instructions. EA.hy926 cells were then seeded onto the coated chambers at 10^4^/well in the fresh assay medium, and incubated for 18 h. The formation of capillary tubes in Matrigel was examined by use of an inverted microscope equipped with a digital camera (Olympus, Tokyo, Japan). The level of the tube formation was quantified by measuring the length of tubes in five randomly chosen fields from each well using an Image-Pro Plus software and subsequently calculating against untreated groups.

### 3.10. Statistical Analysis

Quantitative data are presented as the means ± SEM determined from the indicated number of experiments. Statistical analysis was based on Student’s *t*-test for comparison of two groups or one-way ANOVA for multiple comparisons. *p* < 0.05 was used to determine statistical significance.

## 4. Conclusions

In summary, the PI3K/Akt-Sp1 pathway is involved in resistin-induced VEGF expression in human ovarian epithelial carcinoma cell line HO-8910 cells. This study provides a novel regulatory signaling pathway of VEGF expression induced by resistin, and suggests that antiangiogenesis therapy may be beneficial treatment against ovarian epithelial carcinoma, especially in obese patients.
